# The Hospital. Nursing Section

**Published:** 1902-03-29

**Authors:** 


The Hospital.
Nursing Section.
Contributions for this Section of "The Hospital" should be addressed to the Editor, "The Hospital" Nursing Section, 28 & 29 Southampton Street, Strand, London, W.C.
No. 809.—Vol. XXXI. *SATURDAY, MARCH* 29, 1902.

### Notes on News from the Nursing World.

### INDIAN ARMY NURSING SERVICE.

We understand that Sisters Jessie Oram and A. J. Weighill have been taken on to the strength of the Indian Army Nursing Service temporarily. Miss Oram, who is appointed to the Military Hospital, Rawalpindi, was trained at St. Bartholomew's Hospital, London, and has since 1897 been sister at the Mahommedan Hospital, Cutch Mandrie, India. Miss Weighill, who has been appointed to the Military Hospital, Peshawar, was trained at the Royal Infirmary, Bristol. From 1890 she has been on Lady Roberts' nursing staff, was sister at the Military Hospital, Quetta, Beloochistan, from 1890 to 1891; was attached to the Military Hospital, Sialkote, to 1893; and since April, 1900, has been lady superintendent of Lady Roberts' Officers' Hospital, Murree, India.

### THE WAR NURSES.

The *Nubia* arrived at Southampton from South Africa last week with the following nursing sisters on board:—E. A. Cox, A.N.S., who requires no leave and rejoins the ship; L. G. Kell, A.N.S.R., L. C. M. Noble, A.N.S.R., E. Prangley, A.N.S.R., all time-expired; and H. P. N. Ferreira, civil nurse, requires no leave and rejoins the ship. Five invalided nursing sisters also disembarked, viz., E. J. Martin, A.N.S., J. H. Roberts, A.N.S.R., M. Steensor, A.N.S.R., J. E. Scott, A.N.S.R., and M. T. Neville, A.N.S.R., all on three months' leave.—The *Orotava* also arrived last week with the following on board: M. Hodges, A.N.S.R., requires six months' leave, and will not return to South Africa unless required; M. Wright, A.N.S.R., requires two months' leave and returns to South Africa; E. M. Hill, civil nurse, on six months' leave from Rhodesia, entitled to return passage to South Africa; and M. Housden Gibbs, civil nurse, time expired. The following were invalided home: C. Campbell, A.N.S.R., and Miss Miller, a civil nurse from a refugee camp.

### ROYAL NATIONAL PENSION FUND FOR NURSES.

The annual general meeting of the members of the Royal National Pension Fund for Nurses will be held at River Plate House, Finsbury Circus, E.C., on Thursday, April 24th, at 4 p.m. Any policy-holder of the Fund who wishes to be present will be welcomed by the Council.

### THE PENSION FUND AND GLOUCESTER NURSES.

At the annual meeting of the Gloucester District Nursing Society, held on Friday last week, it was decided to federate with the Royal National Pension Fund for Nurses. It was mentioned that the Society has during the past two years received the sums of £143 and £121 from the Queen Victoria Jubilee Institute in respect of the training of probationers, and that these sums will be set aside as security for the payment by the Society of a moiety of the premiums in respect of policies taken out by its nurses. The annual report records a great increase of work the cases nursed in 1901 numbering 514. There were also 270 midwifery cases and 20,037 visits were paid, as compared with 16,842 in 1900. The Society by its record of good work has attracted the notice of the trustees of the Ossington Nursing Fund and has received a grant of £10.

### SCARCITY OF NURSES AT THE SMALL-POX HOSPITALS.

It is disquieting to have from Mr. Scovell, of the Metropolitan Asylum Board, official confirmation of the reports that there are not enough nurses at the small-pox hospitals. Mr. Scovell, it is true, puts the matter more ambiguously than this. He says that the Board are "not too well provided with nurses in view of the possible requirements of small-pox." But as the figures show that the disease instead of abating, is still creeping on, the managers of the Board ought to be quite satisfied that there already exists ample provision for the nursing. There is no excuse for a scarcity on the ground of lack of means, for an extra penny in the pound has been levied on the ratepayers of the Metropolis in order to meet the demands of the Board. Nor can it be doubted that there are plenty of nurses to be obtained if adequate steps are taken to secure their services.

### PAUPER "NURSES" IN LANCASHIRE WORKHOUSE INFIRMARIES.

A return has been issued by the Local Government Board recording the number of sick inmates in the workhouses of Lancashire, and showing the number of nurses and pauper attendants employed in their care. Of the 16 Unions enumerated, Bolton, Burnley, Chorlton, Haslingden, Oldham, Rochdale, Salford, and Warrington do not allow pauper inmates to assist in the nursing; but the other eight, Barton, Blackburn, Bury, Chorley, Leigh, Manchester, Prestwich, and Wigan do. The condition of affairs in the Blackburn, Bury, and Wigan workhouses is absolutely scandalous. At Blackburn there are 19 pauper assistants to 16 paid nurses; at Bury, 13 to 10; and at Wigan, 16 to 5. Manchester, to its shame, has 14 pauper assistants, as well as 98 paid nurses. In the Leigh and Rochdale unions there is only one paid nurse to every 17 patients, and in Prestwich only one to 18. The publication of these figures is a singular proof of the urgent need of reform in workhouse nursing, even in one of the most go-ahead of English counties.

### AN INDIFFERENT REPORT AT NOTTINGHAM.

Lord Henry Bentinck, M.P., who presided at the annual meeting of the Nottingham District Nursing Association, introduced an interesting personal element in his excellent speech. He told his audience that he himself had recognised the necessity of, and experienced the benefit to be derived from, a trained nurse in case of accident, when he lately broke his leg. This gave point to his criticism of the report, which cannot be called satisfactory. As Lord Henry observed, there is work to be done in Nottingham for 20 nurses instead of 12; and the fact that the receipts, even with an inadequate staff, were £163 less than during the past year indicates a lack of spirit which in a flourishing town like Nottingham was not to be expected. There is no question of the excellent work done by the nurses, who last year nursed 866 cases and paid 30,482 visits: every speaker at the meeting testified to it. Indeed, as one of them said, "the nurses did a great deal more than they were paid for." Of course, the first necessity is to increase the subscriptions sufficiently to cover the cost of the operations of the association, but we hope that, in addition to this, the support will speedily be augmented sufficiently to justify the engagement of more nurses.

### EPILEPTIC PATIENTS AND GARDENING.

The ninth annual report of the Meath Home of Comfort at Godalming for the reception of epileptic women and girls from all parts of the kingdom mentions that, since the opening of the Meath cottage in Hayling Island, which affords accommodation for twelve patients and three nurses, the committee have been able to take an additional number of patients into the Meath Home. In order to relieve the lady superintendent of some of the household management, Nurse Blackith has been appointed to the post of house matron, and in this capacity she undertakes the superintendence of the servants' work. Since last June the services of a lady cook have been obtained; and the introduction of a lady gardener a year ago has so far proved a great success. The latter has now several of the lady patients as pupils in the garden, and this occupation, the report says, is a source of much pleasure to them. It is hoped that in time the garden will be entirely worked by the lady gardener and the patients without any additional help.

### THE SUFFOLK NURSING INSTITUTE.

The principal topic of discussion at the annual meeting of the Suffolk Nursing Association was the gift by Mr. Martineau of a home for the nurses in Ipswich, which he has also furnished at his own expense. It is hoped that by the addition of the home, which is to be called the Suffolk Nursing Institute, the difficulty in obtaining a sufficient supply of nurses for training will be overcome. The nursing under the auspices of the Association was stationary last year. Although three new districts were formed, operations in three others were relinquished in connection with the organisation, so that the number of districts is 25 as before. No explanation was given at the meeting for this action, but we conclude that in two of the cases there was not adequate financial support.

### QUEEN'S NURSES AT BOLTON.

It is a feature of the thirteenth annual report of the Bolton District Nursing Association, that in respect to the 1,534 cases attended by the nurses, almost every case is said to have been "a serious one, actually needing a trained nurse." This shows the importance of insisting upon the employment for district, as well as for other work, of fully-trained nurses. The Bolton staff consists of a superintendent and ten nurses, and we regret to notice that the income last year was slightly less than the expenditure. This is particularly unfortunate at the present moment, because it is necessary to withdraw from investments between £500 and £600 in order to make essential alterations and improvements in the Nurses' Home, and the income will thus be further reduced to the extent of £25. However, if a number of the subscribers will follow the example of Mr. J. H. Halton, who at the meeting said he would, in the circumstances, have pleasure in doubling his usual contribution, the deficiency can easily be made up. There is, it seems, a strong conviction among the subscribers that the collections in the churches and chapels might be on a more liberal scale.

### AN EXPERIMENT IN DISTRICT NURSING.

The first annual meeting of the subscribers to the Eythorne District Nursing Fund has just been held, Lord Guilford presiding. Dr. Duncan read a report of the work done by the nurse, who paid 1,529 visits in the twelve months, of which 570 were to outside villages, involving an average journey of three miles each. The nurse had also attended 22 confinements, and given anæsthetics and otherwise assisted in 22 operations. This result must be particularly satisfactory to Dr. Duncan, who, when he settled at Eythorne, which is the centre of a large and populous district near Dover, found neither nurse nor midwife. He tried to induce the principal residents to interest themselves in supplying the want, but not succeeding at first, he engaged a trained nurse himself at a salary of £40 a year and then called a meeting to approve, or otherwise, the course he had pursued. The meeting was small in number but favourable to the project, and though no committee was formed, subscriptions were promised, the control being left in Dr. Duncan's hands. Since then the subscribers have increased the salary of the nurse to £52, many of the contributions being small sums, and the experiment has been so successful that in three contiguous districts it is now proposed to employ a trained nurse on similar lines.

### A RETROGRADE MOVEMENT.

It is not in the least astonishing that the Atcham Board of Guardians, who have had so many difficulties with their nurses, should have taken upon themselves to initiate a movement having in view the degradation of the status of superintendent nurse at the workhouse infirmaries. They have been pleased to forward the following resolution, which they have passed themselves, to a number of other boards of guardians, asking them to endorse it:

> "That the status of superintendent nurses as provided by the order of the Local Government Board, August 6th, 1897, is so anomalous and unsatisfactory as to require immediate attention and revision. The control by the Guardians is, by this order, completely taken away. They have not even the power to suspend, however serious an offence may have been committed by a superintendent nurse, though this power is possessed by them in the case of the workhouse medical official, the master and matron, and other officials. The result is that proper administration by the Guardians is almost, if not quite, impossible."

In many instances no notice appears to have been taken of the communication, but at Hull it has been approved "in principle," and at Devonport it has been referred to the house committee. It is, of course, of a most retrograde character, and aims at placing in the hands of the guardians additional powers, whereas the powers they already possess are often grossly absurd. If the Dudley Board of Guardians had been legally able to suspend Miss Newbury, it is probable that the scandalous state of affairs she was able to bring to light would have been hushed up.

### CONGRATULATIONS AT WHITEHAVEN.

At the seventh annual meeting of the Whitehaven District Nursing Association, the Mayor congratulated the members that they had fulfilled the aim of the Queen's Jubilee Nursing Association, whose object is to provide a nurse for every ten thousand of population. With a population of 20,000, Whitehaven has its two nurses, who in the year ending February 18th, nursed 125 cases and paid upwards of 6,000 visits. In recognition of their services, which have extended over five years, the committee have awarded them each a bonus of £2 10s. So far as the financial position of the organisation is concerned, the year closed with a balance of £31 16s. 5d. on the right side; but there is one point which is not satisfactory. The subscriptions of Lord and Lady Lonsdale to the association amounted to £85, and those of all the other people in the district to only £86. We agree with one of the speakers at the meeting that the contributions of the Whitehaven folks might be materially increased. It is very generous of Lord and Lady Lonsdale to subscribe so liberally, but their generosity should be more of an incentive to others. The fact that but for their help it would be impossible to maintain a second nurse, somewhat discounts the congratulations with which the Mayor opened his speech.

### PROGRESS IN STAFFORDSHIRE.

The thirteenth annual report of the Staffordshire Nursing Institution shows that the important organisation at Stoke-upon-Trent still continues to make progress. The strength of the staff, of which Miss Shirley is lady superintendent, was in 1901 as follows:—97 private nurses, 18 district nurses, and 28 probationers in training, or 143 in all, as against 141 in 1900. The number of cases received was 883, as against 832; the number of weeks of nursing were 3,878 as against 3,849; and the earnings amounted to £5,994, as compared with £5,871. The balance in credit on the year's transactions was no less than £1,216, of which we are glad to learn £785 14s. 9d. has been apportioned by the committee among the nurses as a percentage on their earnings. It is mentioned in the report that the wisdom of having a reserve fund to meet contingencies has been proved by the call that was made upon it last year to cover the cost of substituting a stone staircase for the damaged wooden one which had for so many years given access to the nursing home from the Newcastle Road, and of strengthening the retaining wall. The accounts appear to be admirably kept, and the institution to be managed in the most efficient manner.

### A CONTRAST.

At a lecture by Dr. Theodore Dyke-Acland given to the supporters of the Hospital Saturday Fund at the Mansion House on Saturday a series of lantern slides were shown, illustrating "Our Fight against Small-pox; or, Common Sense and Vaccination." Among these "contrasts" were two pictures which the lecturer called "The Useless and the Useful Nurse." The useless nurse was one who had refused to be vaccinated. "A nurse," said Dr. Acland, "is no use unless she is at work, and this nurse is in bed and has to have someone to look after her." The face was turned away, but the marks of the disease were painfully evident. The "useful nurse" had, of course, been vaccinated, and was tending a patient with small-pox, looking quite calm and fearless.

### MINISTERING TO THE ORPHAN.

It is pleasant to learn that an orphanage, founded half a century ago on the West Coast of Ireland, in order to save some of the orphans at the time of the famine, is now filled with other children and working happily. Since the Spiddal Orphanage, in County Galway, was erected, the building has been repaired and improved. It receives destitute children of any denomination from any part of Ireland, and depends for support entirely upon voluntary contributions. Miss Smyley and the other ladies who assist her would be cheered and encouraged in their excellent work by a little help from England.

### RATEPAYERS AND SALARIES.

Although there was opposition on the part of certain members of the Chester Board of Guardians to the proposal of the visiting committee that the salary of the superintendent nurse should be increased to £45 per annum, with a further augmentation of £1 per annum up to a maximum of £50, and that of the assistant nurse from £26 to £32, it was eventually adopted. As both these officials are admittedly fulfilling their duties in the most admirable manner, the attempt to raise the cry of "the ratepayers' pockets" was rather ungracious. It is distinctly to the advantage of the ratepayers that the patients in Chester Workhouse Infirmary should be properly nursed, and that there should not be frequent changes in the staff. Morever, since the superintendent nurse was appointed the obligation of training probationers has been imposed upon her. It cannot be pretended that the increase puts her in possession of an extravagant salary.

### A CONVALESCENT DANCE.

The annual ball in aid of the London Hospital Convalescent Home at Tankerton is fixed for Wednesday, April 30th, at the Grafton Gallery. The list of patronesses includes the Duchess of Somerset, the Duchess of Bedford, Consuelo Duchess of Manchester, the Duchess of Sutherland, the Lady Frances Balfour, the Countess of Suffolk, the Countess of Westmorland, the Countess of Aberdeen, the Countess of Portsmouth, the Lady Kathleen Pilkington, the Lady Dorothy Nevill, Lady Tweedmouth, Lady Battersea, Lady Burghclere, Lady Newnes, Lady Lewis, Mrs. Gully, Mrs. Asquith, Mrs. Bischoffsheim, Mrs. George Alexander, Mrs. Alfred Harmsworth, and many others. The ball promises to be a great success, and it is for a very deserving charity. The Grafton Gallery is one of the best places in London for dancing, and the supper arrangements are in the hands of Messrs. Benoist. Mrs. Spender has shown great public spirit in making herself responsible for the entire expenditure of this Home—£800 per annum. We wish her a great success with this dance. Tickets (price £1 1s.) may be obtained from the Honorary Secretaries, Mrs. Spender, 29 Cheyne Walk, Chelsea, and Mrs. Theodore McKenna, 22 Portland Place, W.

### Lectures on Gynæcology for Nurses.

By ROBERT JARDINE, M.D., M.R.C.S., F.F.P. and S.G., F.R.S.E., Senior Physician to the Glasgow Maternity Hospital, Examiner in Midwifery to the University of Glasgow.

### VIII.—AFFECTIONS OF THE VULVA.

*Vulvitis.*—By this term is meant inflammation of the vulva. It may be divided into simple, purulent and follicular, but since the bacteriology of the vulva and vagina has been studied, the infective nature of the complaint has been clearly shown. Various forms of micro-organisms are found to exist in the discharges. In the simple form the predisposing cause is local irritation, which may be caused by dirt, parasites, threadworms, dribbling of urine, acrid vaginal discharges, friction and scratching of the parts. It is very common in strumous children, if they are neglected. It is commonest among the female children of the very poor, and may generally be ascribed to want of cleanliness.

*Signs and Symptoms.*—The parts are red and swollen, with a watery mucous discharge. There is a burning pain when urine is passed, and persistent itching. After a time, if the case is neglected, the discharge becomes purulent. But if the cause is gonorrhœal a muco-purulent discharge shows itself very soon. In such a case there may be eroded patches on the labia and thighs. In these cases there will probably be some feverishness and restlessness at night.

In the follicular form the glands of the vulva are affected, and a number of small boils appear.

*Treatment.*—As the affection is practically always due to the want of cleanliness, the first thing to do is to remove the cause by carefully washing the parts with an antiseptic lotion, and keeping them clean. If pediculi are present, they must be got rid of. In an adult the parts should be shaved, and this will remove the habitat of the vermin. Various ointments of mercury, etc., are used for killing vermin. One of the best is stavesacre. If threadworms are present, they must be cleared out of the lower bowel, which is their lurking place, by giving repeated enemata of salt water. To allay the irritation of the parts, weak carbolic lotion may be used. Weak acetate of lead lotion is also used, or zinc ointment, carbolic ointment, menthol ointment, or an antiseptic dusting powder are employed.

In cases of gonorrhœal infection, appropriate medicines will be ordered by the doctor, but the nurse must be very careful in disinfecting the bed-pan or other utensil used by the patient, and she must also carefully sterilise her hands before touching another patient. A very virulent form of inflammation may be set up in the eyes, if any of the discharge should happen to get into them. The patient must be warned of this, as she may scratch the parts to relieve the itching, and easily convey infection to her eyes. For bathing the parts, clean rags, wool, or tow should be used and immediately burnt.

In the follicular form the contents of the follicles must be evacuated. Tincture of iodine and glycerine, or a silver nitrate solution is often applied.

*Aphthous Vulvitis* occurs in young children generally after fevers. Little white spots appear upon the mucous membrane of the vulva, and they tend to form small ulcers just as they do in the mouth. The same disease is sometimes seen in nursing women. It is caused by the vegetative organism, oidium albicans, which causes thrush in the mouths of children.

*The Treatment* consists in thoroughly washing the parts with an antiseptic lotion, and applying boroglyceride.

*Pruritis Vulvae.*—This is a condition of intense itching of the vulva. It is apt to attack women at the menopause. The itching is so intolerable that the patients can hardly refrain from scratching the parts, and excoriations, which only add to the misery, are apt to be formed. Warmth is apt to increase the itching so that it is generally worse when the patient is in bed. In some cases the suffering is so great that the patient may become irresponsible in her actions and commit suicide. In one of my cases the woman threatened to do this, but fortunately the treatment quickly relieved her or I believe she would have attempted to do away with herself.

The true form of pruritis is really an affection of the nerves and nerve endings. To the naked eye there may be very little change observable except where the parts have been scratched. The same condition of intense itching may, however, be caused by irritating discharges, and may be seen in pregnant women. Diabetes is another frequent cause, so the urine of all women suffering from pruritis should be tested for sugar.

*Treatment.*—As heat aggravates the condition, cold applications generally give the most relief. Alcoholic or ætherial solutions, with 2 per cent. of carbolic or salicylic acid added are sometimes beneficial. Cocaine or opium ointments are sometimes used, and a tampon of ichthyol and glycerine often gives relief. A bran or soda sitz bath is also useful. In cases due to irritating discharges appropriate treatment must be adopted to cure the discharge, and the itching will then cease. In cases due to diabetes local treatment will be of little avail unless the diabetes is treated. To give the patient rest at night sedatives will often have to be administered. When the nerve endings are affected electricity sometimes does good, but in some cases all the affected mucous membrane may have to be removed before a cure can be effected.

*Intertrigo.*—This is a common affection in stout women The external surface of the labia majora which are in apposition to the skin of the thighs, become red and inflamed by friction. The perspiration causes maceration of the epidermis, and an erythema is set up. It may end in eczema. There is considerable itching in the parts, and it may become very painful. The condition is generally associated with want of cleanliness, and is very common where there is a vaginal discharge. In bad cases it may spread for a considerable distance on the thighs.

*Treatment.*—The first essential is absolute cleanliness. After the parts have been thoroughly dried, a dusting powder of starch or rice, with a little boracic or salicylic acid, should be freely used. Astringent solutions as of alum or acetate of lead are also used, and bits of lint soaked in the lotion should be laid between the folds of skin. Various ointments are also useful. If there is a vaginal discharge, it must, of course, be treated.

*Eczema* may attack the vulva and it must be dealt with in the same way as eczema of any other part of the body, by the application of suitable ointments.

*Abscess of the Vulvo-vaginal Glands.*—The vulvo-vaginal glands lie one on either side beneath the labia. Their ducts open just outside the base of the hymen, generally near one of the caruncles. Inflammation of an infective nature may spread up the ducts and cause an abscess of the glands. Both glands may be affected at the same time. A globular swelling forms at one or both sides of the vulva. The pain is generally severe.

*Treatment.*—Hot fomentations should be applied to relieve the pain, and as soon as pus is present a free opening has to be made and the abscess cavity stuffed with iodoform gauze to ensure healing from the bottom. The stuffing must be renewed daily until the wound closes.

An abscess may form in the labia without involving these glands. It has to be dealt with like an abscess in any other part of the body, viz., by free incision and drainage.

*Noma Pudendi* is the name applied to gangrene of the vulva which sometimes attacks young children after fevers. It is the same as noma or cancrum oris which attacks the face. It commences with a burning pain and fever. The parts swell up and become discoloured, and blebs form full of thin serum and sloughing soon takes place. The disease is due to septic infection and is generally fatal. If the patient should survive there will be great deformity from cicatrical contraction.

*Treatment.*—The patient's strength must be kept up by the free use of stimulants and plenty of easily digested food. Locally the affected tissues must be destroyed with the actual cautery, or freely excised and carefully disinfected.

*Erysipelas* sometimes attacks the vulva in young and neglected children, but is also sometimes seen in adults. If it should attack a puerperal woman it will end in puerperal septicæmia and almost surely be fatal. In adults it is liable to assume a chronic form which reappears at the periods and disappears in the intervals.

There is the usual erysipelatous redness of the parts with burning pain and feverishness.

*Treatment.*—Dusting powders of boracic and salicylic acid are useful. Sometimes the part is painted over with silver nitrate, or a hypodermic injection of a 2 per cent. solution of carbolic acid is made. The patient's strength should be kept up with plenty of nourishing food.

*Diphtheria* sometimes attacks the vulva and vagina. It may be primary or more frequently secondary to diphtheria in the throat. It is caused by the same organism as so often attacks the throat. It is sometimes seen in puerperal women, and is then likely to be fatal. The only case I have ever seen was in a puerperal woman.

There is high fever, quick pulse, and prostration, with tenderness in the vulva or vagina. An inspection will reveal the diphtheritic membrane, the same as occurs in the throat. If the attack is a bad one, noma, which we have just described, may occur.

*Treatment.*—The general treatment is, as in other cases of diphtheria, by the use of anti-diphtheritic serum, and the patient's strength kept up with suitable diet and stimulants.

*Tuberculosis* sometimes affects the vulva, and it may also be the seat of *malignant disease. The Treatment* will be surgical, that is, free removal.

*Venereal Sores.*—The labia are the usual seat of venereal sores, but I do not intend discussing them, except to warn you that when dealing with such cases a nurse cannot be too careful to see that she has no abrasions about her fingers, as she may very easily infect herself.

### One Easter Eve.

By a Night Nurse.

Night. The moon was shining "the Paschal moon that shineth brightly" I could not sleep—why? Well, because as you will see, I could not, that was all, and I often wondered how much the outer world realised this life of ours—a night nurse in a hospital ward, to whom the night is as day.

How uncanny this work seems at times, in the small hours of the morning, more especially when one has time to sit down and think. The loneliness of it all—and yet it is not all darkness. To-night, as I said, the moon was shining and it was Easter Eve—and then there is always the day to come; the new day with its new hopes and possibilities. Just now sleep, the kind mother that "takes her tired children in her arms, and they forget," reigned supreme. Even No. 9, had for once a look of peace in her worn face, she also forgot, or wandered afar in the Elysian fields of long ago.

And little Tommy—the pet of the ward—how blissful was his expression, sleeping securely in the belief that anyhow goosey-gander would not come to throw him down stairs as "he did prayed" before he went to bed. He half seemed, however, to hope that the dire fate might overtake less virtuous boys if only he could be awake to see.

There was a box outside which had come for me, but it could not be opened yet. The house physician had not made his round, then there would be the midnight work to do. But when all was quiet again, then a cup of tea, and the box.

"No. 9 holds out" said the house physician a little later, as he made his round, "but it cannot be for long," and he passed on.

Poor No. 9! her's had been a hard life, and many were the bittter words spoken of the man who had wrecked her life, leaving her a deserted wife, so that she had sunk lower and lower in her misery until a worn out body lay on the bed—waiting to be carried out some day.

By 1.30 a.m. the moonlight was flooding the ward, and again all were sleeping and I was alone; yes, alone with thoughts that at this moment turned to tea. Then giving a look round to see that all was quiet, I went softly back to the little kitchen and opened my box.

Oh, how lovely! for before me were daffodils, beautiful golden daffodils and deep purple-scented violets; the daffodils telling of the golden day that had dawned, and the violets so redolent of perfume in their dewy freshness.

Easter Day, and we are told that "the women very early brought sweet-smelling spices." They were not wanted then, but now those sick and suffering ones for whose sake the joy of the first Easter morning dawned should have an offering too. So hastily vases and bowls were filled and put round on the lockers by the bedsides of the patients while they slept, and on the table, where all could see them, so that the awakening eyes might be gladdened and their hearts filled with the message the flowers would bring, "Emblems of our own great resurrection."

Then I left the blossoms while I went to prepare the breakfasts. No. 9 should have a special cup of tea in a special cup and saucer. It should be a day of days for her.

And ere I had finished my work, delighted whispering came from the ward, and told me that the night was over, and that the fresh breath of spring had aroused the sleepers.

The church bells were ringing the Easter hymn as I brought No. 9 her tea. There she lay, some of the flowers in her hand, and she seemed caressing them fondly, with something like a far away smile on her worn face.

"Eh! nurse! but they're good," said she, as I came up. "It seems like as 'twere long ago once more." (This was much from No. 9, who had rarely spoken before without bitterness, nor even acknowledged anything done for her.) "Aye," she went on, as if to herself, her eyes on the flowers, "I mind it was Easter. Me and Jim we was married, and the daffodils was out; but now, where is he?" and the tears coursed slowly down her cheeks.

"Why?" I wanted to say, "you said you never wished to hear of him again." But I held my tongue.

"Yes, where is he?" she continued. "Why, nurse, 'tis me that would crawl twenty miles on my knees just to see him again."

Love is eternal, and the shadows were falling.

Then, having finished her tea, No. 9, turning away, lay quietly, a wistful expectant look upon her face.

They all seemed happy at their breakfast; the windows were open, the Easter sun shone in, and as the soft wind from the outside played in among the daffodils, little Tommy cried, "Why, nurse, I thinks they're laughing."

And when night came again No. 9's bed was empty.

### Six Months' Private Work.

### A NURSE'S PERSONAL EXPERIENCES.

(Concluded from page 332.)

### A Case of D.T.

After that I had one good night's rest, and next day I was sent to a case of delirium tremens at a large hotel. On my arrival I found a very sad state of affairs, as the proprietor was raving in one room with this awful complaint, and his wife, for whom I had been fetched, was nearly as bad in another. Her brother and his wife received me, and were very kind indeed, doing all in their power to make me comfortable, and my patient also seemed to be more tractable than I had dared to hope for. She kept me pretty busy all night, however, but in the morning was so much better that I had time to think of her husband and make inquiries as to his condition. They told me he had been very violent during the night, but that the doctor had been and injected morphia, and that he was now sleeping. They had two men to sit with him, and as they seemed perfectly satisfied I had no wish to interfere further. My patient remained in bed, and about 3 P.M., feeling very tired, I laid down on the couch in her room. I had just dozed off to sleep when I was aroused by the sister-in-law, asking if I would come to the husband, as they did not like the look of him. I went at once, and was horrified to find him in a state of collapse, with stertorous breathing. I took his temperature, which was 107·4°, and sent someone post haste for the doctor. As he only lived a few doors away he was quickly on the scene. As he had been away his locum tenens had been taking his place and this was the first time that the doctor had seen the man. He was confident that I had made a mistake in the temperature and took it himself, when it registered 107·6. There was no doubt that the poor man was dying fast and although we did our best for two hours trying restoratives, he never rallied and died at 6 P.M., his wife meanwhile being fast asleep and perfectly unconscious of all that was going on.

### A Transformation.

When she awakened we had finished our sad task for the dead. Her brother broke the news to her very gently, being fearful for the result, but it seemed to have a calming effect and perhaps nothing could have sobered her more effectually. I was astonished at the extraordinary transformation in her, for, from a hysterical invalid, she became at once a resolute, calm, and business-like woman. The whole household seemed thoroughly disorganised by the suddenness of the blow that had fallen upon them, save the widow. She seemed at once to step back into her rightful position of mistress of that large establishment and nothing escaped her or was forgotten. My post seemed quite a sinecure, but as I was asked to remain I did so and helped with domestic matters, for several friends and relatives were expected for the funeral and rooms had to be prepared for them. My ex-patient might have been a wooden figure, so calm and quiet was she over the shopping and other details. To me was deputed the task of fetching home the two children from school, and their grief was extreme. They had evidently worshipped their father, and, poor mites, they refused to be comforted. Next came the funeral, to which I was bidden in my capacity of nurse and companion to the widow, but to my surprise I was put more in the character of a mourner, going in the first carriage with the chief mourners. Except for a few tears when her children clambered into the carriage, the widow never gave way, at least in my presence. I had not many idle minutes, for I often had to preside at impromptu meals to visitors on business, etc., and the remainder of my stay was not altogether unpleasant, but I was taken suddenly with influenza and the doctor ordered me home at once, greatly to the disgust of my lady superintendent, for it was the busy season, and I could ill be spared. However, I was obliged to submit to fate, and being really ill was only too thankful to get home and go to bed, where I remained for three days feeling more dead than alive.

### "An Urgent Summons."

On the fourth day I received an urgent summons, and feeling very weak and giddy I managed to drag myself back to the Home to find that the superintendent wished to send me at once to fetch a patient from Norwood, and convey her down into Norfolk that day. Needless to say I protested, for I felt far too ill to do anything save to go to bed again, but I pleaded in vain. She only laughed in my face, and told me that if I was not able to do as she told me now I need not do so any more, as she wanted people who could work, not invalids. So there was nothing for it but to pack my boxes, leave them to be sent on, and then return home, and to bed, where I was obliged to remain for three weeks, having taken a fresh chill after the influenza. When I came back to convalescence, the sight of my luggage reminded me that my first spell of private nursing was at an end.

### Presence of a Nurse Resented.

I began to think also that my few months of private work had not been altogether a success, but taking heart of grace, I determined to do a bit more if possible, and with this object in view I interviewed one or two doctors for whom I had worked, and they promised me some of their cases. About a week after this I received my first summons, and departed to a town a few stations off. The patient, a gentleman, had been ill for nearly three years with rheumatoid arthritis. It was a sad case, and one where all one's care and attention seemed in vain. The poor fellow must have suffered agonies, and yet through it all he was very patient, bearing his terrible sufferings with a sublime patience. He was very well off, and the house was very nice, but his wife was not so sympathetic as she might have been. She had, it appeared, greatly resented the idea of having a nurse in the house, chiefly, I think, on the score of expense, for she was the very meanest woman I have ever met. She seemed to begrudge us the food we ate, and I was very glad I had not a large appetite, as in that event I should have been nearly starved. I had a hard job to get her to procure the necessary nourishment for her husband, and she had a most irritating habit of coming upstairs after his meals and asking if he had enjoyed a certain thing "because if not he ought to, for it had cost so much"—mentioning the price. This of course had a most disastrous effect. Then, after I had been there a few days, I saw that the wife was growing jealous of me; she refused to do anything for her husband's comfort, and didn't seem to like the idea of anybody else doing it. She began to throw out vague hints of getting a housekeeper and nursing her husband herself, so I could plainly see I was not wanted, and, taking the initiative, gave the date of my departure. My poor patient begged of me to remain, but I knew it was useless to try to do any good against so much opposition, so I reluctantly bade him farewell. When I heard of his death, three months later, I could only feel glad and thankful that his sufferings were over. So ended my six months' private work, for after this last case I was so thoroughly run down that I was ordered a long rest, and determined at its close not to tackle any more private patients. I was not sorry for my short experience, and am not going to say in *Punch's* immortal words, "Don't," to anyone who intends going in for similar work, for I think that the change and experience is good; but as a permanent thing I shall never recommend going in for private nursing. It may, perhaps, be said that I was particularly unfortunate in my cases, for I have met many nurses who regard private work as the only nursing worth doing when one has finished training, but *chacun à son goût*, and mine is for the regular, if hard, work of an institution.

### A Scheme for Affiliated Hospital Nursing.

By a Former Hospital Matron.

That there is often a difficulty in supplying our smaller hospitals, both in country districts and in some of the smaller towns, with the right material to form an efficient nursing staff, must be evident to anyone who studies the advertisement columns of The Hospital from week to week. Probationers are being asked for in numerous cases. Some of these show the difficulty by offering "baits" to catch the evidently not too numerous candidates, such as "a certificate at end of year," or "two years," "age from twenty," "no premium," etc. And one sees the same hospital advertising again at the end of a few months.

### The Cause.

The reason is not far to seek. It is not so many years since the training given in one of these hospitals was considered sufficient to prepare a nurse for private nursing or for a post of greater authority in another hospital. Then, too, many of these smaller institutions found willing workers who gave their services for the sake of helping their sick and suffering fellow-creatures. Some few of them are being still supplied in the same way by devoted women who work without pay, and in one instance known to the writer pay is given to the hospital for this privilege. But this is confined to the very few, and the majority of young women of to-day who are aspiring to enter the nursing profession think more of the value of their training and the certificate it confers than of the mere nursing of the sick poor. This is not unnatural, for, since nursing has become a profession, and largely since private nursing has become so lucrative that the public demand the most highly trained nurses in return for the large fees required by them, the nurses feel that they cannot afford to "waste their time" in working for a certificate which, from this point of view, is of little value. Nor do they care to work for one or two years in one of these small hospitals, gaining much useful experience and doing much good work, only to be told on applying to one of the larger hospitals that "candidates who have worked in other institutions are never received here."

### The Smaller Hospitals.

Now no one can deny that these smaller hospitals, containing from ten to eighty or ninety beds, are doing an excellent work, nor that much excellent experience, from a nursing point of view, can be obtained in them. The charitable people who have given, and now support these most useful institutions expect, and have a right to demand, that the sick poor who benefit by them should be efficiently and skillfully nursed in them. The funds will not allow of sufficiently large salaries being paid to fully trained and certificated nurses; nor, indeed, will fully-trained nurses do much of the work which is necessary in the wards—work which is beyond the province of a wardmaid and which is now properly performed by a probationer nurse. How to procure the most suitable probationer is a subject which causes much thought on the part of the matron, and often she spends many of her leisure moments in devising schemes to attract and keep the most suitable nurses. Yet frequently she has to take in those candidates who, on account of height, strength, or it may be through lack of great advantage of education, have been unable to find entrance into a big London hospital. At the same time she feels that, though unable to give a complete course of training, she can yet offer an excellent training in the practical details of ward-work and nursing, and can also promise an extremely good foundation in theoretical teaching in the first-class courses of lectures given by the able house physician or surgeon. Such a training, in fact, as our large hospitals are beginning to find is needed as a preliminary to their full curriculum, and to attain which one or two have already started elementary training schools, such as those at Glasgow and at the "London" Hospital.

### A School of Preliminary Training.

All our large hospitals cannot afford to do this, though no doubt they would profit by some such school of preliminary training. Now this object might be accomplished by a well worked-out scheme of affiliation between the nursing departments of the smaller and the large hospitals to the advantage of both. Groups of small hospitals might be thus affiliated (for nursing purposes only) with a large general hospital in London, or in the provinces, if over 100 beds; the matron of such large hospital drawing a certain number, if not all, her probationers from those nurses who have passed successfully through the course of training laid down at these smaller hospitals. The course of this preliminary training should be settled by a joint conference of the matrons and committees of the large hospital and its affiliated branches, and it should be terminated by an examination in theoretical and practical work done, the successful probationers passing on to the large hospital for its full course. The probationers could be taken into the preliminary hospital at an earlier age, say at twenty to twenty-two, binding themselves for two years with the understanding that, if successful, they would at the end of that time pass on to the full course of training at the head or large hospital. This would practically mean a five years' course, but I feel sure that nurses would willingly undertake this first course if they were only certain by such means of securing entrance into the large general hospitals, especially if the age were lower than that required for entrance into the latter. Thus the supply of nurses could be kept up for the small hospitals, where matrons could then be able to make a judicious selection of the most suitable candidates from the larger number of applicants that this scheme would attract. If the sisters of the branch hospitals were drawn from those nurses who received their preliminary training in the same hospital and had gained their certificate in the affiliated larger one, it would he an added incentive to good work and would also draw more suitable candidates to the hospitals of smaller size.

### Possible Objections.

No doubt objections would at first be raised to this affiliation scheme, especially by those authorities who still hold the somewhat old-fashioned idea that to make a good nurse she must come quite fresh and ignorant of all nursing knowledge to the training school of the hospital she will eventually work in. But surely the matrons of the smaller hospitals are no less fitted to train probationers than they were when sisters of the very hospitals who often now reject their nurses? Probationers entering at an earlier age could more readily afford to give their services for these two years in return for board, lodging, uniform, training, etc., and this would allow of the salaries of the sisters being raised to a sum more in accordance with the value of their services.

### To Nurses.

**We invite contributions from any of our readers, and shall be glad to pay for "Notes on News from the Nursing World," or for articles describing nursing experiences, or dealing with any nursing question from an original point of view. The minimum payment for contributions is 5s., but we welcome interesting contributions of a column, or a page, in length. It may be added that notices of appointments, entertainments, presentations, and deaths are not paid for, but that we are always glad to receive them. All rejected manuscripts are returned in due course, and all payments for manuscripts used are made as early as possible after the beginning of each quarter.**

### A Nurse's Experience as Stewardess.

By an Occasional Correspondent.

So many nurses have inquired through the correspondence columns of The Hospital about the post of stewardess, how to obtain it, and what the duties are, that my actual experience of the post may be interesting to those who wish to try it.

### Few Vacancies.

I am very fond of the sea, and had been four long pleasure voyages before I decided on trying to get a berth as stewardess. I started by applying to every steamship line that carried passengers, except the American, and the answer was always the same: "we have more applications than we have vacancies for, and they are always filled by relatives of those who have died in our service." At last, through personal influence, I obtained the wished-for berth, on a Cape boat, but it was only for one trip.

### The Work.

The work of a stewardess varies on the different lines, but on all of them the essential qualifications are good temper, patience under all circumstances, brightness, and neatness. These all trained nurses should possess, so that there is no reason why they should not do well in such posts; but on every side I heard the same thing: "trained nurses stand so much on their dignity, and will only do such things as they think are their proper work." It is a great pity, but it is the same thing on shore; people often dread having a trained nurse in the house, because she wants so much waiting on.

### Pay and Uniform.

The pay, of course, is purely nominal. The company that I worked for gave a uniform, consisting of a navy blue serge dress and one cap, apron, collar, and pair of cuffs. This was always to be worn while in the home port. In warm climates one could wear what colour print or cotton dress one liked, and always, of course, spotlessly clean aprons and collar and cuffs. Anyone thinking of getting a berth as stewardess, should be well provided with clothes, as washing at all foreign ports is ruinous, the usual charge being 4s. a dozen, and many of the washerwomen have not the slightest idea of getting up a nurse's cap or strings, the frills being simply ironed straight out.

### "Fore-Cabin" Stewardess.

I rather dreaded the ordeal of the Board of Trade Office, but in reality it was not at all unpleasant. I had to "pass the doctor," but this was purely a matter of form, and then a clerk read a paper which I could not hear, and I had to sign my name on what I suppose were the ship's articles, and it was done. I was second class, or, in ship's language, "fore-cabin" stewardess. We were to sail at four o'clock on Saturday, and I had to be on board at 11 A.M., and on my arrival I was given a list of my passengers, with the numbers of their cabins. I had 25 ladies and 26 children, the youngest of whom was one month old on the day of sailing. My duties were to wait on the ladies in their cabins, superintend the children's meals, make the lower berths (ladies') and fill their water bottles. When there is a young baby on board, and the lady has no nurse, one naturally offers to take it for her occasionally, and if there are no ladies in bed during the day to take it while the mother has her meals, as, of course, a baby is never allowed in the saloon.

### The Day's Work.

Briefly, a day's work is as follows. Rise at six o'clock, take tea and coffee to all who wish for it, and find out those who wish for baths. Then get sufficient bath towels from the linen-room steward, afterwards call the children and their nurses, because, unless the ship has a children's saloon, they have to have their meals before the adults. While they are dressing, send the ladies to the bath-room in turn, putting in the steam to make the water hot for those who require it, passengers never being allowed to turn on the steam themselves. After that the children have their meal, at which the nurse has to be present. The serving of their meals is generally left to the boy stewards, and they are apt to be very careless and inattentive. After breakfast the making of beds, etc., starts, and all the cabins have to be in perfect order by 11 A.M. and oneself spick and span ready for inspection by the captain, first officer, doctor, and chief steward. Following lunch, till 4.30, everyone rests, the stewards taking it in turns to be on duty in the saloon, in case anyone wants anything or a bell rings. The day ends at 10 P.M. I always used to go round to all my ladies and say good-night, and see that they had all that they were likely to require before I went to bed; the result was that I was never sent for after I had retired.

### Neither Hard nor Unpleasant.

The first two days at sea are the worst; after that everybody settles down, the majority get over their sickness, and all prepare to enjoy the beauties and delights of an ocean trip. Of course, if the ship puts into various ports, one is apt to get more or less busy, and there are necessarily disagreeables, but the work on a well-regulated steamer is neither hard nor unpleasant, and I am quite sure from what I have seen that a great part of a stewardess's troubles are of her own making. As far as I am concerned, if I could get a berth as a permanency, I would gladly go to sea again. I met with unqualified kindness from all on the ship and I know that the passengers appreciated the comfort of having a trained nurse as stewardess, and were very grateful for any little personal service which I was able to render them.

### An English Nurse Killed near Mount Lebanon.

A correspondent from Beyrouth writes: A terrible accident, involving the death of two nurses and a patient, has just happened near Areyah Station, Mount Lebanon. Miss Alice Wood, in charge of the Friends' Hospital, Brumana, accompanied by a native nurse, went to take a patient back to her village, and on the way the carriage overturned and fell down a precipice, all three being killed. The two nurses were hastily placed in roughly-made coffins, brought back to Brumana, and buried together in the same grave in less than 24 hours after the catastrophe. The father of the native nurse is a schoolmaster in the Friends' School, and all the inhabitants of the village, stricken with horror and grief, came out to meet the coffins. They all attended the funeral, although of many and varied religions.

Before the coffins, which were smothered in flowers, were conveyed from the meeting house to the grave, the father of the native nurse addressed the mourners as follows: "I am proud to think that my daughter was a nurse, and thankful to hear, on all sides, of the testimony of her kindness and devotion to the patients, and of her good and pure life, and this, I know, is greatly owing to the good influence of the lady who left her parents, home, and country, to come here and devote her life to the nursing of the sick of this country, and whose body lies in this other coffin, but whose spirit is now with God."

Miss Alice Wood, to whom this touching tribute was paid, was trained at Tunbridge Wells General Hospital, and came out to Brumana nearly three years ago. She was deservedly popular among all classes, and the tokens of grief, which were manifested at the funeral, were hearty and sincere.

### Everybody's Opinion.

[Correspondence on all subjects is invited, but we cannot in any way be responsible for the opinions expressed by our correspondents. No communication can be entertained if the name and address of the correspondent are not given as a guarantee of good faith, but not necessarily for publication. All correspondents should write on one side of the paper only.]

### HOW TO MAKE HOSPITAL MEETINGS ATTRACTIVE.

"Sister Ellen" writes: I have been much interested in reading accounts of the annual meetings of various London hospitals recorded in your valuable paper, and noticing the paucity of members attending, in some cases scarcely sufficient to form a quorum. I consider this apathy on the part of subscribers a great loss to the hospitals, as a personal visit will often create an interest and induce the subscribers to increase their subscriptions, also allowing them to see and hear how the money is expended. I feel sure that if the various officials of the respective hospitals would make a more attractive meeting beside the reading of accounts many people would gladly avail themselves of the opportunity of visiting the hospitals on such occasions. The hospital over which I have the honour of being matron had similar uninteresting meetings until I induced our committee to provide tea and make an "at home" of the day and throw the wards open to inspection, with the result of an attendance of between 200 and 300 friends who take a deep interest in the institution, both by contributions and personal sympathy. After such functions we receive many additional subscribers, so that the trouble incurred is amply repaid by increase of funds. All hospital committees have one plea—lack of funds—that any suggestions for raising such should, and I believe can be obtained.

### NURSING IN BRITISH COLUMBIA.

"A Nurse in Vancouver" writes: Perhaps some of your nurses would like to hear about my travelling out here. I came out in September, bringing a baby of three and a-half years old, to his parents who reside at Esquimalt. We had rather a rough passage, and I, myself, was seasick, and unfit for much for five days. Luckily my baby charge had a very slight attack, but being not very strong, it pulled him down, especially as it was followed on landing by the six days' train journey, with very little exercise. Never, though, shall I forget the glorious scenery of that journey, such beautiful blending of nature's colours one can never describe. Sometimes we stopped for half an hour, and I found the negro attendants most obliging to myself and baby, always giving us warning when to "get aboard." Here you are bound to watch, as there is no calling out as at our English stations. Going up the "Rockies," the dining car—where the food is very good—was taken off, as it is too heavy to take up the mountains, therefore we had nothing to eat from 6.30 P.M. till 11.30 A.M. next day. When we got out, we went to the most perfect little hotel on the platform, and were waited on by Chinese waiters. On arriving at Esquimalt with baggage and baby charge at 9.45 at night, it was rather overwhelming not to be met, after travelling thousands of miles alone. Also owing to the telegram announcing our arrival not having reached, there was no supper ready, no fire burning, no beds prepared, and the child's parents were out to dinner. I did indeed wish myself back in "Home, sweet Home." There was only a Chinaman to greet us, and when I asked for Mrs. W. he said, "I not know, she go out, come home bye-and-bye," and left us alone to meditate on our happiness. I truly hope no nurse will ever feel as I did on my arrival. Now that I have settled down, although far away from England, one does not realise the distance because the majority of the people here are English. It is curious to see Chinamen carrying their goods in baskets on either side by means of long poles round their neck. We have a great many Chinamen here. Our Chinese cook is very funny. He thinks the English nurse does "too much talk, too much hard work." Here there is a splendid "field" for English trained nurses, and the salary is good, from 30 to 40 dollars a month. A dollar is 4s. 2d. in English, but the Canadians have a great contempt for money. A dollar to them is no more than our shilling is to us, and their smallest coin is 2½d. They have no copper coinage. The climate here as yet has not been much colder than "Home," but by and bye we shall get rather colder weather and a very little snow. Though I am so "far away" I am always thinking of "home," and I long for my Hospital, but here one cannot get English nursing literature.

### "A QUESTION OF ADMINISTRATION."

"Lady Superintendent" writes: Does a hospital derive any real advantage from the attachment of a ladies' committee? The answer requires some consideration, and consideration that can only be fairly given by one who has had practical experience of a hospital without a ladies' committee and a hospital with a ladies' committee. The object, as far as the public is concerned, is, that more interest may centre in the hospital when the wives of the provincial magnates bestir themselves to visit the institution. That more interest does centre in it is an undoubted fact. That the ladies in a small town possess facilities for securing annual subscribers to the hospital fund, is also a fact. That they could, if so minded, provide many small extras for the benefit of the patients, is also possible. That they could help the lady superintendent to procure, without incurring hospital expenditure, many ward necessaries, such as dressing gowns, shoes, jackets, and many other things, is probable. All these charming things—and more—a ladies' committee could do with advantage, and were these things to come to pass, lady superintendents and sisters all over the land would "rise and call them blessed." Unfortunately, the list of their deeds (or misdeeds) is frequently noticeable for a marked deficiency in philanthropy, and an equally marked desire to interfere with business not within their province, and distinctly out of range of their knowledge. The ladies on the committee are generally married. They have the inestimable honour of presiding over their husband's establishments; they sometimes even have such a vast charge as the care of two or three servants. Their experiences, gained in so "large" a manner, must necessarily be of advantage to mankind, or more especially to womankind—at least so they think. They burn to give forth their ideas. They thirst to implant their views on the lady superintendent, and try to impress upon her the fact that the methods which they employ in managing a household of six persons must necessarily be the best methods to apply to the responsible charge of 100 people. That the unfortunate head of the hospital has been trained to the work is, of course, absolutely unimportant; the ladies care nothing for that. The possibility of having a finger in someone else's pie is too alluring to be resisted, and once their fingers are in, they want to stir the pie round, Then the "last state of that hospital is worse than the first." Speaking reasonably it is most difficult for private ladies to understand the methods of public administration. Their environment has not fitted them to deal with daily life on a large scale; their ideas are grooved in things "small," and if a woman has common sense she will recognise this and refrain from interfering with the administrative part of a public institution. That the interference of a ladies' committee does harm is a certain fact, and a fact that has been proved many times in the past history of hospitals. The effect of their visits can be plainly seen in the domestic part of the establishment; a marked irritability in the manners of the nursing staff—especially the sisters—and a simmering dissatisfaction in the servants' domain can be plainly felt, and these elements do not tend towards the successful working of any hospital. Disciplined women, who are trained to deal reasonably and justly with the patients in many trying situations, naturally chafe and rebel at suddenly being confronted with an absolutely unreasonable element surrounding their own lives, and which materially interferes with the efficient discharge of their duties. Looked at from a practical point of view it is not too much to say that the drawbacks arising from the formation of a ladies' committee connected with a hospital far outweigh the few advantages that are ever likely to accrue from the increased interest of a few outsiders.

### The Nurses' Bookshelf.

The Practical Nursing of Infants and Children. By Frank Cole Madden, F.R.C.S. (Cassell and Co., Limited. 1901. Pp. 287. Price 3s. 6d.)

There are many people who, knowing no more about babies than the average yeomanry recruit does about horses, imagine that they are qualified to speak on the subject of the medical and surgical treatment of infants. But there are others, with little better credentials, who venture into print on the same subject, and this is a much more serious matter, for readers into whose hands these literary productions may fall, are not always in a position to judge of the merits of the work, or the qualifications of the writer. These strictures on authorship are not, however, in the least applicable to the case of the book which lies before us, for it has been written by a competent authority on the diseases of children, who has had the invaluable experience which attaches to the post of medical superintendent of the Great Ormond Street Hospital for Sick Children, and subsequently that of a hospital surgeon in Cairo. As regards the general design and arrangement of this little work, we have nothing but praise to offer, while the manner in which common-sense views are combined with technical knowledge is only what would be expected of a writer with the practical experience of Mr. Madden. It must be remembered, however, that the author addresses his remarks more to nurses in hospitals and public institutions than to mothers or domestic nurses who have received no special training. And for this reason a considerable portion of the book which describes the preparation of patients about to undergo important surgical operations, and the management of fractures and dislocations, will be found altogether unsuitable, except for those who are about to become, or are already, trained nurses. The probationer class of nurses will, however, doubtless appreciate the practical manner in which the nurses' duties in the wards are explained. And the fact that the sections which deal with surgical, medical, and infectious cases have been submitted to the criticism of experienced hospital sisters will be at least some guarantee that theoretical do not outweigh practical considerations.

With reference to the chapter which describes the artificial feeding of infants, it is noticeable, and perhaps to be regretted, that none of the modern methods of milk modification are mentioned, with the exception of that which is the basis of Gærtner's system. This method, however, is not often employed in this country, and further it is open to the objection that this variety of humanised milk must necessarily be submitted to a process of centrifugalisation. Mechanical interference of this kind with the physical properties of milk is now generally recognised as undesirable, however accurately the chemical composition of the milk may simulate that of the mother, and it is this objection, which is equally applicable to the percentage of milk preparations, supplied by milk laboratories, which have brought all milks of this kind into some degree of disrepute. We consider, therefore, that Mr. Madden's work would have been more complete had he described in detail some method of milk modification which is free from this objection. We notice also that no description is supplied with regard to the administration of emetics, or to the induction of emesis. This is one of the most useful expedients in the treatment of bronchial affections of children, and in the emergencies which attend indiscretions of diet. Knowledge with regard to this subject certainly belongs to the province of the professional nurse. With the few exceptions to which we have referred, we believe that this little book will be found by probationers and nurses to contain all the information they are likely to require from books with respect to the surgical and medical duties in the wards of a children's hospital.

The Lemco Cookery Book.

The Lemco Cookery Book is a handy little volume containing 180 recipes compiled by Mrs. H. M. Young, in nearly all of which Liebig's extract of meat finds a place. This serves to show how useful Lemco is in the kitchen, and there is no doubt that in many households it will be found to be quite a stand-by. We believe that Messrs. Liebig are willing to supply their useful little cookery book gratis to anyone desiring it. Their address is 9 Fenchurch Avenue.

### Appointments.

Bromsgrove Union Infirmary.—Miss Florence Baker has been appointed superintendent nurse. She was trained for three years at Stockport Union Infirmary, and was subsequently charge nurse at Bury Union Infirmary and Walsall Union Infirmary.

Chelsea Hospital for Women.—Miss Lilian Bramston has been appointed charge nurse. She was trained for 18 months at the East London Hospital for Children, and for three years at the General Hospital, Birmingham. She has since been for four years in a London surgical home, has done six months' temporary sister's duties at Poplar Accident Hospital, and has been assistant nurse at Chelsea Hospital for Women from December, 1900, to the present time.

Fulham Infirmary.—Miss Susan Mary Meredith has been appointed sister. She was trained at Paddington Infirmary for three years, and for six months as midwifery pupil. She holds the L.O.S. certificate.

Hitchin Union Infirmary.—Miss F. Ewbank has been appointed superintendent nurse. She was trained at the Salford Royal Hospital; has been private nurse for two years at the Victoria Home, Bournemouth; charge nurse for four and a half years at the Hahnemann Home, Bournemouth; and charge nurse for three years at Ecclesall Infirmary, Sheffield.

Lanchester Union Infirmary, Durham. — Miss Gertrude Walker has been appointed superintendent nurse. She was trained for three years at the Royal Infirmary, Bradford. She has since done six months' district work, 15 months' private nursing, and has been charge nurse at the Infirmary, Lanchester.

Native Location Hospital, Capetown.—Miss M. M. Goy has been appointed matron. She was trained at the National Hospital for the Paralysed and Epileptic, Queen Square, Bloomsbury, and Lincoln County Hospital, and was one of the twenty nurses who left England for plague duty in Capetown last April.

Suffolk General Hospital.—Miss Constance Hodges and Miss Emma Spencer have been appointed sisters. They were both trained at Addenbrooke's Hospital, Cambridge.

### Presentations.

Ecclesall Infirmary, Sheffield.—Miss F. Ewbank, who has been charge nurse at the Ecclesall Infirmary, Sheffield, for the past three years, has been presented by the doctors and nursing staff with a silver mounted writing case. Miss Ewbank is leaving Sheffield to take duty at the Hitchin Infirmary as superintendent nurse.

The Mount Vernon Hospital for Consumption, Hampstead and Northwood.—At a special meeting of the house committee held at Mount Vernon Hospital, Hampstead, last week, the chairman, Mr. B. A. Lyon, presented to Miss Whitton, on behalf of the board of management, a cheque for £50 together with a handsome dining-room clock on her retirement from the matronship of the hospital. During Miss Whitton's tenure of office the number of patients has largely increased, and in making the presentation Mr. Lyon alluded in terms of praise to Miss Whitton's ability and devotion to duty during the four and a half years she had been with the committee.

### Echoes from the Outside World.

### Movements of Royalty.

The King will spend a few days of his cruise in the Solent off Cowes, sleeping on board his yacht, and afterwards he will probably proceed down Channel. As the tour is to be strictly private, should the yacht put in at any port there will be no formal reception. At the Council at Marlborough House on Monday, the King signed a proclamation making the Coronation Day and subsequent day Bank Holidays.

The Prince of Wales performed the ceremony of opening the new National Physical Laboratory at Teddington on Wednesday last week. He was accompanied by the Princess. The idea of the establishment of the laboratory originated with Lord Kelvin, who when he was President of the British Association in 1871 urged the importance of such an institution. It was not, however, until 1897, when a Treasury Committee was appointed, that the movement was seriously taken in hand. This committee having reported to Parliament in 1898 in favour of founding a public institution for standardising and verifying instruments, for testing materials, and for the determination of physical constants, it was decided to wind up the old Kew Observatory Committee and to vest its powers and properties in a newly-elected National Physical Laboratory Committee, who are chiefly Fellows of the Royal Society. Ultimately the Government offered to assign to the Royal Society an old Royal residence, Bushy House, with a grant of £14,000 to remodel it, and a maintenance allowance of £4,000 a year. As the Prince of Wales, in his inaugural speech pointed out, the laboratory must largely rely upon private generosity for its equipment; and, as a matter of fact, several gifts have already been received.

### South Africa.

On Monday it was announced that Mr. Schalk Burger, the Acting President of the Transvaal, with Mr. Reitz and Commandants Lucas Meyer and Krogh had arrived at Pretoria by special train from Balmoral under a flag of truce. It was also intimated that the Boer leaders not finding Lord Kitchener at Pretoria, proceeded south of the Vaal in order to meet him. Questioned in the House of Commons, the Secretary for War said that a fortnight ago Mr. Burger expressed to Lord Kitchener a desire to be permitted a safe conduct through our lines and back, in order to see Mr. Steyn with reference to the possibility of peace proposals. Lord Kitchener, he added, "with the assent of His Majesty's Government," acceded to the request.

Mrs. Fawcett, president of the Concentration Camps Commission, addressed a large meeting on Friday last week respecting the work of the Commission in South Africa. She gave a detailed account of their method of inspection. With a prepared list of what they wished to ask, they called first upon the superintendent of the camp, put questions to him and took notes of his answers. They then separated into groups of two, each group taking a particular department for inspection. After seeing the superintendent they said "good-bye" to the officials, unless they wanted to ask them any special question as to the camp. The members of the Commission, Mrs. Fawcett stated, went about the camps entirely unaccompanied, observing for themselves. They also visited people in the tents separately, because they felt that women were more likely to speak openly if visited by only one person. There were complaints, some reasonable, and others unreasonable, but there was not a single complaint of the conduct of our soldiers, nor of the manner in which the Boer prisoners had been treated. The tents of the Boer women were indescribably dirty, as may be judged by the fact that one young woman, being trained by a doctor as a probationer, was asked by him when she last had a bath. She replied "Two years ago," and, as may be imagined, she was sent off to have one at once. Mrs. Fawcett mentioned that among the delegates from the Continent was a lady representing the Queen of Holland, who confessed that she found things very different from what they had been represented to be.

### Philanthropic.

A great number of the friends of the Royal Normal College for the Blind at Norwood were present at the annual meeting last week, held, by the courtesy of the Duke and Duchess of Sutherland, at Stafford House. In the absence of the Archbishop of Canterbury, the chair was taken by the Earl of Aberdeen, who spoke of the wonderful manner in which the pupils at the College are trained. He had visited the institution himself, and found it almost impossible to believe that the happy-looking inmates who walked about unaided in so natural a manner had been deprived of their sight. They were taught self-reliance in every way, and the principal, Dr. Campbell, might be proud of the fact that out of those educated at the College 89 per cent. now earned their own living. Mr. J. H. Choate, the United States Ambassador, said that there were 50,000 totally blind people in America against 30,000 in England, and both countries educated on much the same lines. He alluded to a blind deaf and dumb girl, Helen Keller, who, notwithstanding her infirmity, entered the American College, which corresponds to Girton, and has gained first-class honour in advanced Greek. He had such confidence in Dr. Campbell's energy and determination that he was sure if he lived he would obtain the £15,000 for which he was striving to pay off the existing mortgage on the Norwood buildings. To help in this good object a concert is to be given in Queen's Hall on April 25th, when purses will be presented to the Prince and Princess of Wales. Both before and after the meeting on Thursday the pupils rendered several vocal pieces in thoroughly finished style.

Thanks to the efforts of Our Dumb Friends' League, a society for the encouragement of kindness to animals, an ambulance for horses has been constructed and was on view last week. In appearance it closely resembles the vans used for the conveyance of prize stock, but there are certain distinct features, notably a stretcher, 4 feet 6 inches wide, having rollers beneath. This is well padded and provided with straps. The idea is that a horse, having been incapacitated, the bed is placed alongside the animal and it is then turned over and secured, the bed containing the horse being subsequently hauled up the inclined plane into the ambulance by a windlass, which is affixed at either end of the ambulance for the purpose. A criticism of experts is that the bed is not sufficiently wide, but the reply is that an animal can be accommodated on it if its legs are folded, and that a wider vehicle could not be legally used in the streets of London. At all events, it will be hoped that this distinctly practical attempt to provide succour for wounded horses will meet with success.

### The Art of the Day.

The show of the Royal Society of British Artists is quite up to the average. Sir Wyke Bayliss has a fine drawing of Amiens Cathedral, and among the more striking works in the central room are "Wild Clouds of Destiny," by Mr. G. H. Lenfestey; several landscapes by Mr. R. Vicat Cole; "Stapleton Vale, near Bristol," by Mr. F. A. Armstrong; a portrait of Mr. Orchardson, R.A., by his son Mr. Charles Orchardson, and of Professor James Stuart, by Mr. Leonard Watts. In the gallery devoted to water-colours, Mr. T. F. M. Sheard's study, "An Interested Observer," Mr. Lenfestey's "Gay Life on the Lido," and Mr. Spendlove's "Morning in Tuscany" are three of many excellent contributions.

The Royal Institute in Piccadilly has been painted and cleaned and the water colours show up the better for the renovation. There is not this year a very large percentage of noteworthy pictures. Mr. H. J. Stock has a delicately painted portrait of "Lady Penrhyn," Mr. Arthur Severn a charming sea-piece "Sunset at Sea, Cumberland Coast," and Mr. Dudley Hardy in "Fishwives, Boulogne," shows how much care and trouble an artist may successfully take in painting ugly models.

### National Sport.

The fifty-ninth annual boat race on the Thames between Oxford and Cambridge Universities took place last Saturday, Cambridge winning by five lengths. This is a very easy victory, and though it was generally expected that Cambridge would be triumphant, it was not anticipated that the rival crew would be so badly beaten. Oxford has still many races to the good, the record of victories being Oxford 33, Cambridge 24, and one dead heat. As usual, the contest took place in far from pleasant weather, but the crowd in the vicinity of Putney Bridge and all along the banks of the river to Mortlake was nearly as great as ever.

### A Book and its Story.

### MISS MARY JOHNSTONE'S NEW NOVEL.*

Miss Mary Johnstone is a born story teller; she has also an admirable literary style, with a keen perception of historical colouring and accuracy of detail. These traits are particularly noticeable in her new book "Audrey," and make it a worthy successor of her previous works, "In the Old Dominion," and "By Order of the Company," which made her reputation on both sides of the Atlantic.

The scene of this last story is laid in Old Virginia, when George the First was King, and my Lord Orkney, as his representative, held office as Governor-in-Chief. It is a good old-fashioned romance, with plenty of adventure and thrilling scenes of love and rivalry, and others which give space for the display of less strenuous emotions. In the background is Audrey, little Audrey, the child of the woods and mountains, with her delicate elf-like beauty and simple passionate nature, living in a world of dreams, dreaming in a world of realities, through whose moods the dramatic temperament was stirring, which later was to bring all the world to her feet when, as an actress, she found expression for the gift that was hers. Living in the solitudes of a Virginian forest, she is first made aware of the world beyond by a column of horsemen, gay and debonnair gentlemen, bent upon sport and exploration, who ride into the valley where stands her poor cabin home.

Marmaduke Howard is one of the party. A young man of fortune, contemplating a voyage to England, and a stay of some length in the gay world of London before settling down on his estate. "He was young; he could spare the years. One day he would come back to Virginia to the dear old garden and quiet house. His factor would give account, and he would settle down in the red-brick house, with the tobacco to the north and east, the corn to the west, and to the south the mighty river—the river that lay just beyond him, gleaming through the trees." An injured ankle necessitate his falling out of the line of his mounted comrades, and he found himself left behind a few miles distant from the cabin in the valley. With his slave, Tuba, he rode slowly on through the forest that seemed to have no ending. His thoughts were fixed on the distance, and he saw there "the face of a girl he had seen in the valley, shy and innocent, the face of the woodland maid who had fixed his fancy, who was drawing him through the wilderness back to the cabin." "What if the cabin, the sugar-tree, the crystal stream, had sunk from sight like the city in one of Monsieur Galland's fantastic tales? Perhaps they had done so, the spot had all the air of a bit of fairyland, and the woodland maid had gone to walk with the elves. Perchance for her it would be better so. And yet it would be pleasant if she would climb the hillside now, and sit beside him with her shy dark eyes and floating hair. The night would not then be so slow in going." Suddenly the mountains are lit up with the reflection of a red light burning fiercely down in the valley below. "It is the valley that we have been seeking, Tuba," said his master, speaking rapidly and low. "That burning pile is the cabin, and 'tis like that there are Indians between us and it." They hasten forward to come upon the charred ruins only of the pioneer's home. Silence reigned. No sign of life was present. A shadow of something lying upon the ground startled them. It was Audrey who sprang up—the only survivor of the happy home party—upon whose seclusion Howard had broken earlier in the day. Her story was a heartrending and tragic one. The Indians on the warpath from north to south had come down and murdered the inmates, and set fire to the valley cabin. Her life was spared owing to her hiding-place not having been discovered. "The face of the child was dark and thin, but the eyes were large and there was promise in the mouth. He looked at her again and suddenly resolved that he, Marmaduke Howard, would provide for her future. He would take the child to Williamsburgh with him, and get some woman to tend her until he could get kind and decent folk with whom to bestow her. There were the new minister of Fairview parish and his wife, they would do. Oh, she should be well cared for. He would—if he thought of it—send her gifts from London; and when she was grown, and asked in marriage, he would give her for dowry a hundred acres of land."

He placed Audrey with Minister Darden and his wife, "kindly, bluff, downright folks," as he believed, and having so far done his duty to his little charge, he thought little more of her during his absence in England. Upon his return, after ten years—years not without episodes and experiences—he was still very much the same man as when he set out to see life. An enigmatical, complex personality difficult of comprehension to outsiders, "he had many selves, and each in its turn, the scholar, the man of pleasure, the indolent, kindly, reflective self—took its place behind the mask and went through his allotted part. His self of all selves, the quiet, remote, crowned, and inscrutable I, sat apart, alike curious and indifferent, watched the others, and knew how little worth the while was the stir in the ant hill." Of marriage, as an accessory to his position, he had entertained the idea more than once; but his wooing of the fair daughter of the owner of an adjoining estate was not received by her with favour. She realised that the union, from his point of view, was merely one of *convenance*, and, as such, she, loving Howard passionately, would have none of it. Other women had failed to win him, and the great house at Fair Mount remained without a mistress. Audrey, growing up a fair wild flower in the social desert that surrounded the home in which she had been left by Howard, was victim to the hated attentions of a half-bred Indian trader, who frequented the Minister's house, and was his boon companion. Fleeing into the woods to find sanctuary she comes one day to the boundary of the glebe and Fair View lands, and encounters Howard. He finds upon inquiry that she is unhappy and neglected. She does not at first recognise her benefactor until he speaks of the mountains and the sugar tree and the little valley among the hills, and then "She turned upon him, with her soul in her eyes. For her the years rolled back . . . the air was filled with the trampling of horses, the sound of a trumpet . . . between the rows of rustling corn at night time, and in terror, she felt again the clasp of her pursuer, and heard the comfort of his voice. 'Audrey,' said Howard, 'come here, child.' Howard, looking at her, watching the sensitive, mobile lips, reading in the dark eyes, beneath the felicity of the present, a hint and prophecy of love, felt for her a pity, so real and great, that for one moment his heart ached as for some sorrow of his own. To Audrey this was a very perfect knight, a great gentleman, good and pitiful, that had saved her from the Indians when she was a little girl, and had been kind, oh! so kind, in that dreadful night when she had lost father and mother, brother and sister, when in the darkness her heart was a stone for terror he had come like God from the mountains and straightway she was safe. Now into the woods from the over-sea he had come again, and at once the load upon her heart, the dull longing and misery, had lifted. . . . The glamour was at hand, the enchanted light which breaks not from east or west or the north or the south was on its way; but she knew it not, and she was happy in her ignorance." Onward to the end the interest quickens and grows until the story reaches its tragic dénouement.

* "Audrey." By Mary Johnstone. (Publishers: Constable and Co., London. 1 vol. Illustrated. Price 6s.)

### For Reading to the Sick.

EASTER-TIDE.

Springtide birds are singing, singing,
For the daybreak in the East,
Silver bells are ringing, ringing,
For the Church's glorious Feast.
Christ is risen! Christ is risen!
Sin's long triumph now is o'er.
Christ is risen! Death's dark prison
Now can hold His Saints no more.
Christ is risen! risen, Brother!
Brother, Christ is risen indeed!

. . . . . . .

Christ is risen! Christ the Living,
All His mourners' tears to stay,
Christ is risen! Christ forgiving
Wipes the stain of sin away.
Christ is risen! Christ is risen!
Sin's long triumph now is o'er;
Christ is risen! Death's dark prison
Holds His Faithful never more.
Christ is risen! risen, Brother!
Brother, Christ is risen indeed!

*From the Greek.*

At Eastertide one thought must ever be present to our mind—the remembrance of those who "sleep in Jesus." We think with gladness of Christ's Resurrection power, strong to save us from the misery of sin; but we never can forget at Easter the other victory of the Lord—the victory over death. "Christ the first fruits; afterward they that are Christ's at His coming."

How could we bear this ugly wrench of death, how could we ever smile or play, or love again, "if in this life only we had hope"? Most of us have some sacred spot where lie the relics of our dearest treasure. To-day we go to the quiet resting-place of our sacred dead, and as we look at the spring flowers preaching to us so sweetly of the great renewal, we whisper softly, "In the likeness of His Resurrection." One by one they will be given back to us, "conformed to the body of his glory." Here they were "in the likeness of His death," girt about with all the frailty and sore distress which comes alike to all, sharply pressed by anxious toil and bitter pains: "My flesh and my heart faileth," often enough they cried. But soon they will awake and sing, brilliant they will come forth in the unapproachable and unknowable splendour of the risen Christ, understanding "all mysteries and all knowledge," fulfilled with the abundant sweetness of the love of God. The same familiar form, the same gentle, playful smile, the same tender heart, only perfect with all the majesty of the risen life. "Thanks be unto God which giveth us the victory through our Lord Jesus Christ."

*T. B. Dover*, "*Alive unto God.*"

The graves grow thicker, and life's ways more bare
As years on years go by:
Nay! Thou hast more green gardens in thy care,
And more stars in thy sky!

Behind, hopes turned to griefs, and joys to memories
Are fading out of sight:
Before, pains changed to peace, and dreams to certainties
Are glowing in God's Light.

No jubilees, few gladsome festive hours
Form landmarks for my way:
But Heaven, and earth, and Saints, and friends, and flowers
Are keeping Easter Day! *R. E. Y. A.*

### Notes and Queries.

**The Editor is always willing to answer in this column, without any fee, all reasonable questions, as soon as possible.**

**But the following rules must be carefully observed:—**

**1. Every communication must be accompanied by the name and address of the writer.**

**2. The question must always bear upon nursing, directly or indirectly.**

**If an answer is required by letter a fee of half-a-crown must be enclosed with the note containing the inquiry, and we cannot undertake to forward letters addressed to correspondents making inquiries. It is therefore requested that our readers will not enclose either a stamp or a stamped envelope.**

*Home.*

(230) Will you kindly tell me where a man of 27, in the last stage of consumption, would be well cared for for the rest of his life? A sum of from one to two pounds could be paid weekly by his friends.—*M. B.*

The Friedenheim Home of Peace for the Dying, Sunnyside, Upper Avenue Road, Swiss Cottage, N.W., has four beds for paying patients at the fee you name.

*Supplementary Training.*

(231) Could you tell me where nurses, with two years' training, are received, and where, for an additional year's probation, a three years' certificate is given?—*Anxioue.*

It can hardly be expected that an institution of good repute will undertake to certify that a nurse has had three years' training when, as a matter of fact, it has only been responsible for one. The Local Government Board in some cases recognise a certificate from two different schools as equivalent to one of continuous three years' training, but it is necessary to ascertain that the schools in question are so recognised before entering the second for supplementary training. The advantages of a three years' certificate are so great that it would be worth while to try and see if some recognised school would not engage a holder of a short time certificate as assistant nurse, at the same time extending to her, in recognition of a contract for three years' service, the privilege of instruction (when required), examination, and full-time certificate.

I have had nearly three years' fever training, and would be glad if you could tell me of any hospital where probationers are received for less than three years who have had previous training. —*L. M. B.*

See reply to "Anxious." Fever training is valuable as an addition to general training, but it is not a substitute for it.

I understand that there are certain hospitals where supplementary training is given. Could you tell me which they are?—*Iso.*

The City of London Hospital for Consumption and Diseases of the Chest, Victoria Park, E., is the only one which has made provision for this, but it is a special hospital, and therefore can only be of service to such as have had training in general nursing. See reply to *Anxious.*

*Sanitary Inspector.*

(222) Can you oblige me with any information how to qualify as lady sanitary inspector? I am a trained nurse. Can I be prepared for examination by a postal course of instruction?—*Nurse Ena.*

Apply to the secretary, the Sanitary Institute, Margaret Street, W., or of the National Health Society, 53 Berners Street, W., for particulars and advice.

I am anxious to qualify as factory inspector. Will you kindly tell me how I can do so?—*Anxious.*

Apply to the Factory Department, Home Office, Whitehall, S.W.

*Crêches.*

(233) Can you kindly give me the names of some "crêches" in England, and tell me where I can get rules and particulars for organising the same?—*M. H.*

Dr. Barnardo, 18 and 26 Stepney Causeway, London, E., might be able to help you.

**Standard Books of Reference.**

"The Nursing Profession: How and Where to Train." 2s. net post free 2s. 4d.
"Burdett's Official Nursing Directory." 3s. net; post free, 3s. 4d.
"Burdett's Hospitals and Charities." 5s.
"The Nurses' Dictionary of Medic l Terms." 2s.
"Burdett's Series of Nursing Text-Books." 1s. each.
"A Handbook for Nurses." (Illustrated). 5s.
"Nursing: Its Theory and Practice." New Edition. 3s. 6d.
"Helps in Sickness and to Health." Fifteenth Thousand. 5s.
"The Physiological Feeding of Infants." 1s.
"The Physiological Nursery Chart." 1s.; post free, 1s. 3d.
"Hospital Expenditure: The Commissariat." 2s. 6d.

All these are published by the Scientific Press, Ltd., and may be obtained through any bookseller or direct from the publisher, 28 and 29 Southampton Street, London, W.C.

### Travel Notes.

**By Our Travelling Correspondent.**

### XCVI.—SPRING IN NORTH HOLLAND.

If it can be avoided do not take an autumn holiday in Holland, the spring is the pleasant time there, and as there are but few trees one misses the glory of the autumn tints, which are so lovely in a more wooded country. The lush meadows and straight poplar-edged roads are at their best in spring and early summer, so too are the effects of misty mornings on the canals when the bright sun breaks through and gilds everywhere the quaint houses with their gabled and staircased roofs and the low arched bridges constructed only to allow of the passage of the barges.

### Amsterdam and Immediate Neighbourhood.

I shall tell you of the chief points of interest in North Holland—Friesland and Groningen, all of which may be seen in a fortnight—but I do not approve of such rapid sight-seeing, which savours too much of the customs of our trans-atlantic cousins, who think nothing of "doing" Rome, Naples, Florence, and Venice, with a few minor towns thrown in, within the space of three weeks. If your holiday is limited to a fortnight, my advice would be to remain in North Holland, leaving Friesland and Groningen for another year.

Amsterdam should be your starting point, and it is so admirably situated as a centre for seeing the country that I almost think it is advisable to remain there all the time, if economy is an object It would however be preferable, if circumstances admit of it, to spend half the time at Alkmaar or Hoorn, but you must always remember that moving your quarters means increased expense. There are such delightful ways of getting about—rail, trams, and steamboats—that you can see everything easily from Amsterdam, and not sleep away a night, unless in visiting the Helder, which is a place of doubtful interest to the majority.

### Amsterdam Itself.

The great point of interest is the picture gallery, but of this I must not speak much, or I shall have room for nothing else, and almost any guide-book will give you all necessary information. To understand and appreciate what a wonderful city Amsterdam is, it is necessary to bear in mind that it is entirely built on piles driven deep down through shifting sand to a bed of thick clayey mud. It is computed that the Royal Palace alone rests on over 13,000 piles.

The city consists of 90 islands connected by canals; hence it is called the Dutch Venice.

The busiest spot almost is the Dam, a large square, where are the Exchange, the Royal Palace and the Nieuwe Kerk. From here most of the principal streets diverge (the city is crescent-shaped), and here, too, is the tram centre.

The Exchange is modern and uninteresting, and the Royal Palace is coldly and austerely magnificent, but not very pleasing; it was once the Town Hall, and was better suited to that use than to be a domestic building. The royal apartments are fitted up in First Empire style; the yellow tea-room and small dining-room are fairly comfortable, but the large dining-room and the splendid reception-room, 100 feet in height, chills one with the white and glaring grandeur and utter absence of all snugness, if one may use so plebeian a word with regard to the royal residence. There is a tower to the palace which affords a grand view, I am told, but I have never climbed it, and consider it fatigue and money thrown away.

The Nieuwe Kerk is really fine, but the Dutch Protestants have contrived to spoil most of ther fine churches, and one misses that indescribable reverent feeling which seems to permeate the buildings dedicated to the Roman Catholic faith. Here, where there should be a high altar, is a monument to Admiral de Ruyter. There is some very fine stained glass descriptive of the raising of the siege of Leyden in 1574.

The Jewish quarter is very curious and should be visited, if possible, on a Friday; almost anything can be bought there, but the chief articles of commercial enterprise are marine stores in all their branches. The Jews are very numerous in Amsterdam and have several synagogues. Not far off is the Jodenbreestraat. No. 4 is the house where Rembrandt lived for eight happy years with his wife Saskia van Ulenburgh. The same year that saw his greatest triumph given to the world, "The Night Watch," brought his heaviest sorrow, and his much loved Saskia was taken from him. This wonderful picture is in the Rijks Museum, so arranged by means of a shaded place in which the spectators sit, as to throw up to the best advantage its wonderful effect of light and shadow. Rembrandt is buried in the Wester Kerk. The Rijks Museum is so marvellous for the treasures that it contains that I think you will be fain to spend two or three mornings there.

### The City Charities.

The Dutch manage their public charities very well, and I believe I am right in saying that there are in Holland no workhouses. There is the Protestant Asylum for aged poor in the Binnen Amstel, and the Hospice of St. James for Roman Catholics, both admirably managed. The Blind Asylum, open to the public on Wednesday, is well worth seeing. No charge is made, but you are expected to buy some trifle made by the inmates. Orphanages abound, and the children look rosy, happy, and in excellent condition. The Society for Public Welfare was founded in 1784 by a Baptist, and its chief object is the improvement of the lower classes.

### Modes of Getting About.

Tramways and steamers take you everywhere at very small expense. Monnikendam for Marken is reached by tram, and so is Edam, and steamers go to Alkmaar (a delightful excursion) and to the Helder. The Helder—only to be visited if you have plenty of time—is the extreme point of North Holland at the junction of the Zuyder Zee and the North Sea, and close to the island of Texel. You can go the whole way by the canal to Helder—time occupied about seven hours—and return the next day by rail to save time. It gives you an excellent idea of the country and of the wonderful system of canal travelling.

Try also one morning to go by boat along the North Sea Canal which connects Amsterdam with the ocean, and enables even large vessels to reach the city. The Dutch are a dogged people, as one realises when one sees the marvellous dykes, again and again constructed and destroyed, by which they maintain their footing against the hungry sea, and by studying the Polders, which, once unwholesome morasses, have by immense labour and expense been so reclaimed that they have become the most fertile and rich of the Dutch pastures.

---

### TRAVEL NOTES AND QUERIES.

To Rome by the Riviera (Sunflower).—This is a very pleasant way in the spring. Stop at Avignon, Nice, San Remo, and Genoa, then go either by Pisa or Florence, whichever suits you best. Fares, first class, £10 3s. 4d.; second, £7 1s. 8d., allowing 17 days to be spent on the journey. If you wish to be longer on the road, it is quite easy to make special arrangements. The cheapest route is *viâ* Newhaven and Dieppe: first class, £8 15s. 6d.; second, £6 2s. 10d. As to clothes, light woollen is the safest and most convenient. Washing dresses are not very well got up, and the cost is large in Italy. Remember to take a light wrap with you when visiting churches in Rome: however warm it is outside, there is a dangerous change inside. The same peril lies between the sunny and shady sides of the street.

